# The Effect of Opioid Abatement Funds on the Utilization of Medications for Opioid Use Disorder

**DOI:** 10.1111/1475-6773.70173

**Published:** 2026-11-03

**Authors:** Jiajing Scarlette Shi, Hefei Wen

**Affiliations:** ^1^ Department of Public Management and Policy Andrew Young School of Policy Studies, Georgia State University Atlanta Georgia USA; ^2^ Jimmy and Rosalynn Carter School of Public Policy, Georgia Institute of Technology Atlanta Georgia USA; ^3^ Department of Health Policy and Management Emory University Rollins School of Public Health Atlanta Georgia USA

**Keywords:** medications for opioid use disorder, opioid abatement fund, opioid use disorder, supplantation policy

## Abstract

**Objective:**

To evaluate whether opioid abatement fund allocations to treatment programs influence the utilization of medications for opioid use disorder (MOUD).

**Study Setting and Design:**

The study employed a synthetic difference‐in‐differences design and a triple‐differences model using a state‐year panel to estimate the effects of abatement fund spending on MOUD utilization. The study examined both general treatment allocations and MOUD‐specific program funding.

**Data Sources and Analytic Sample:**

Data on state opioid abatement fund allocations were obtained from the Kaiser Family Foundation tracker. In 2022–2023, 33 states allocated funds to general treatment and 31 to MOUD‐specific programs. Treatment variables were defined as binary indicators of whether a state allocated any funds to these categories. The primary outcome was MOUD‐related admissions from 2016 to 2023, obtained from the Treatment Episode Data Set‐Admissions. Outcomes were measured as substance use treatment admissions per 100,000 population, MOUD admissions per 100,000 population, and MOUD admissions as the proportion of all substance use treatment admissions.

**Principal Findings:**

Spending in the general treatment category led to a 13.2% increase in overall substance use treatment admissions (*p* = 0.06), but not with MOUD utilization. In states prohibiting supplantation, defined as prohibiting the use of abatement funds to replace existing opioid program expenditures, both general treatment and MOUD‐specific allocations significantly increased MOUD admission rates by 232.7% (*p* = 0.009) and 157.7% (*p* = 0.07), respectively. No significant effects were observed in states allowing supplantation. Among individuals with opioid use disorder, allocations on both general treatment and MOUD‐specific programs in no‐supplantation states significantly increased substance use treatment admissions.

**Conclusions:**

General treatment investments modestly increase overall substance use treatment utilization, but effects on MOUD utilization were not detectable in the early implementation period. Exploratory analyses suggest larger effects in states prohibiting supplantation, though these estimates rest on few comparison states and require further confirmation.

## Highlights

**What Is Known on This Topic:**

- Since 2022, opioid abatement funds have been distributed to states and local governments, with at least 70% designated for opioid remediation activities.
- States and localities retain discretion over how funds are allocated, resulting in substantial variation in spending priorities and supplantation policies across states.

**What This Study Found:**

- Allocations to general treatment programs increased overall utilization of substance use treatment services.
- States prohibiting supplantation of abatement funds experienced significantly larger increases in MOUD utilization than states allowing it, though these estimates rest on few comparison states and require further confirmation.

## 1 Introduction

In the United States, the opioid crisis has affected an estimated 6.1 million individuals with opioid use disorder (OUD) [1], a condition characterized by a harmful pattern of opioid consumption that results in severe clinical impairment or distress [2]. OUD is associated with substantial reductions in quality of life, lowered productivity, experienced mental disorders, and elevated risk of overdose and infectious diseases [3, 4, 5, 6, 7].

Medications for opioid use disorder (MOUD) remain the gold standard for OUD treatment. MOUD includes buprenorphine, methadone, and naltrexone, which are evidence‐based treatments that alleviate withdrawal symptoms and support recovery. Studies have demonstrated that MOUD lowers recidivism rates and mortality when provided in correctional settings [8]. Buprenorphine and methadone are associated with reduced heroin use [9], all‐cause and overdose mortality, and opioid‐related acute care [10, 11], and decreased engagement in risky behaviors associated with infectious disease transmission [12]. Despite strong evidence, MOUD remains substantially underutilized, with fewer than 20% of individuals with OUD receiving these medications [13]. Key barriers to MOUD access include limited availability of buprenorphine in pharmacies [14], regulatory restrictions on methadone [15], and stigma toward MOUD as replacing one opioid with another [16].

In response, states have implemented a range of policies to expand treatment access, including removing prior authorization requirements for buprenorphine, adopting Medicaid Section 1115 waivers, and broadening Medicaid coverage for MOUD [17]. However, persistent financial and workforce constraints continue to limit the scalability of these interventions [18]. Federal funding streams, such as State Opioid Response grants, have supported state efforts but are often time‐limited and administratively constrained [19]. As a result, states may face challenges sustaining a long‐term response to the opioid crisis.

The emergence of opioid abatement funds presents a critical opportunity for states to strengthen their response to the opioid epidemic. Following extensive litigation, major pharmaceutical manufacturers and distributors have agreed to pay over $50 billion in opioid abatement funds to states up to 18 years, resolving lawsuits over their role in the opioid epidemic [20]. Opioid abatement funds are governed through state‐specific legal agreements, legislation, and administrative structures that determine how funds are allocated, who has spending authority, and how expenditures are monitored. Some states place the majority of funds under the control of a state‐wide abatement fund or council, whereas others allocate most funds directly to state agencies or local governments [21]. Moreover, the nationwide opioid settlements established strict regulations and required that at least 70%–85% of these funds be allocated for opioid remediation programs and interventions [22]. Opioid remediation programs encompass care, treatment, or other programs and expenditures intended to address opioid misuse and abuse, treat or mitigate opioid related disorder, and address other harms associated with the opioid epidemic [22]. These requirements were informed by lessons learned from the 1998 Tobacco Master Settlement Agreements, under which limited spending oversight led to funds being diverted to fill state budget deficits or unrelated infrastructure projects, with less than 3% spent on smoking cessation programs [23]. Despite the establishment of oversight structures, advisory committees, and efforts promoting public input and transparency, states and localities still retain substantial discretion over how opioid abatement funds are managed, allocated, and reported [21, 22]. This discretion has generated debate over which activities should qualify as appropriate opioid remediation investment, for instance, whether and to what extent opioid abatement funds should be spent on law enforcement [24]. In 2022–2023, approximately $6 billion in abatement funds were distributed to state and local governments [25]. Based on publicly available tracking data for this period, treatment for opioid use, including the expansion of MOUD as an evidence‐based intervention, was a major area of funds allocation, accounting for 18% of reported expenditures, while other purposes of programs each accounted for less than 15% [26, 27, 28]. For example, Alameda County in California launched an innovative mini‐grants program that prioritizes funding for local projects that expand access to MOUD and engage underserved or disconnected individuals in opioid treatment [29, 30].

A key concern regarding the use of opioid abatement funds, especially amid ongoing uncertainties in federal funding, is whether these funds may replace rather than supplement existing state expenditures on opioid‐related programs. This practice, known as supplantation, is not explicitly prohibited under many opioid abatement agreements [24, 31, 32]. For example, lawmakers and treatment advocates in New York have raised concerns that the governor's proposed budget substitutes opioid abatement funds for a portion of the state addiction agency's baseline funding [33]. Such supplantation may undermine the intended expansion of resources brought by the abatement funds and reduce the net investment in addressing the opioid crisis [32]. Accordingly, public health experts and officials have recommended strict non‐supplantation policies as the best practice. In 2021, the Johns Hopkins Bloomberg School of Public Health recommended that jurisdictions adopt legislation prohibiting the use of opioid abatement funds to supplant existing funding streams [25, 34]. However, adoption of such restrictions has been uneven. According to the Kaiser Family Foundation (KFF), only 13 states (Connecticut, Delaware, Kansas, Maine, Maryland, Massachusetts, Michigan, Nevada, New Jersey, New York, Vermont, Virginia, and Wisconsin) and Washington DC currently impose non‐supplantation provisions related to opioid abatement funds [24]. Enforcement mechanisms typically rely on monitoring and regular reporting requirements to ensure compliance with spending rules, including supplantation restrictions [31]. In addition, states such as Connecticut and New York have implemented more explicit enforcement approaches. For example, Connecticut requires annual verification that opioid abatement expenditures are at least equivalent to prior‐year funding levels [35].

Given the substantial financial resources dedicated to addressing the opioid crisis, it is critical to assess whether opioid abatement funds are effectively achieving their intended policy goals as states and local governments allocate these resources across programs and jurisdictions. Emerging empirical literature has begun to evaluate the impact of abatement funds on health outcomes, including opioid overdose deaths, and finds evidence of associated reductions [36]. However, variation in spending decisions may translate into differences in program effectiveness [22]. For example, prior work shows that states with greater fiscal and overdose‐related need tend to spend more abatement funds per capita, suggesting that resources are, at least in part, directed toward higher‐need areas [37].

More evidence is needed to understand whether abatement funds are effectively translating into improvements in key dimensions of the opioid response, particularly MOUD utilization, given its documented underutilization and prominent role in opioid abatement spending. This study addresses this gap by providing causal evidence on the effects of opioid abatement funds on MOUD utilization. In an additional analysis, the study examines whether these effects vary according to state‐level supplantation policies, providing insight on how the fiscal design of abatement funds may shape their effects.

## 2 Methods

### 2.1 Data Sources

This paper linked several datasets to form a state‐year panel from 2016 to 2023. First, the study used the KFF tracker to obtain information on states' opioid abatement fund spending for 2022 and 2023 [20, 29]. The KFF Health News, the Johns Hopkins Bloomberg School of Public Health, and Shatterproof disseminated a survey by the end of year 2023 asking states' plan to track abatement fund spending [28]. Guided by professional suggestion to address the opioid crisis, most states have categorized the purpose of the funds into four buckets and nine core strategies [38]. The four buckets include prevention, treatment, recovery, and harm reduction [28]. Specifically, the Treatment bucket is defined as “Medication‐assisted and nonmedication addiction treatment, including counseling and related efforts.” The nine core strategies include one that specified MOUD distribution and other opioid‐related treatment [38]. This strategy on MOUD includes distribution of medications such as buprenorphine and methadone, provide inpatient and outpatient treatment integrating medication and counseling, and provide education on MOUD to healthcare providers and other first‐responders [38]. The KFF tracker reports the total opioid abatement funds received by state and local entities in year 2022 and 2023, based on information collected from state government websites and the website of BrownGreer, the court‐appointed settlement administrator [28]. Further, the KFF tracker categorized funds spent or committed among states and local entities into the four buckets and nine core strategies based on their definitions and activities. The tracker published by KFF was independently reviewed and shared with states to ensure accuracy and transparency [28]. The data analyzed in this study were collected by KFF and partners as of August 2024 and reflect government actions as of December 31, 2023 [28].

Second, the study used the Treatment Episode Data Set Admissions (TEDS‐A) to measure the outcome of MOUD utilization at the state level. TEDS‐A is maintained by the Substance Abuse and Mental Health Services Administration [39] and collects information on admissions to state‐licensed and publicly funded substance use treatment facilities. The dataset is an administrative dataset and includes state identifiers, demographic information for each admitted individual, the reason for admission, and their prior substance use disorder (SUD) treatment history.

Lastly, the study included state‐level covariates to account for potential endogeneity. This includes the total population and number of Medicaid beneficiaries at the state level obtained from the University of Kentucky Center for Poverty Research [40], the number of mental health and substance use treatment facilities at the state level from the Census County Business Patterns datasets [41], states' prior authorization prohibition status on buprenorphine obtained from literature [42], and states' Medicaid expansion status under the Affordable Care Act (ACA) [41, 43]. The study obtained state's supplantation status in 2022–2023 from the KFF report on supplantation [24, 44].

### 2.2 Study Cohort

This study established a state‐year cohort from 2016 to 2023 which contains the admissions to substance use treatment facilities, states' population, and related policy information. While TEDS‐A collects information from all states annually, several states have missing data for some years. States were excluded if they have incomplete data between 2016 and 2023. The excluded states are Arizona, Delaware, Idaho, Oregon, South Carolina, Montana, Oklahoma, Virginia, and West Virginia, leaving 42 states in the analysis. The final state‐year panel includes 42 states across 8 years, representing 336 state‐year observations.

#### 2.2.1 OUD Sample

To test the effect of the OUD population who are more likely to be influenced by these funds, the study constructed an additional analytic sample by restricting admissions to users who reported opioid as their primary substance used from the tertiary primary substance use variables, including heroin, non‐prescription methadone, and other opiates and synthetics [45]. Individuals reporting these opioids as their substance use serve as a proxy for those experiencing OUD, consistent with previous literature using TEDS‐A [45].

### 2.3 Measures

#### 2.3.1 Treatment Variables

The study defined treatment variables to capture two levels of implementation of the abatement funds on MOUD utilization using the KFF spending tracker. These treatment variables are (1) States' funds committed or spent on the general Treatment bucket, and (2) States' funds committed or spent on MOUD services or programs defined in the core strategy [28]. These treatment variables were coded as binary and measured as whether the state or any sub‐state level entities committed or spent any funds during 2022–2023. Because the KFF tracker does not distinguish between funds committed and funds spent or report allocations separately for 2022 and 2023, this study uses allocation and expenditure interchangeably and treats reported funding as applying equally to both years.

#### 2.3.2 Outcome Variables

The outcomes of interest are the total number of SUD admissions and the number of admissions involving the use of MOUD from the TEDS‐A dataset. An admission is classified as involving MOUD if opioid medications, such as methadone or buprenorphine, are included in the client's treatment plan.

Outcome variables are measured as rates of SUD and MOUD admissions per 100,000 state population, as well as the proportion of SUD admissions that involve MOUD. Because the rates of SUD and MOUD admissions are skewed, the analysis uses log‐transformed versions of these variables.

#### 2.3.3 Supplantation Status

Using the KFF report on states' supplantation status [24], the study constructed a binary variable measuring states' supplantation status: states that prohibit supplantation of abatement funds on existing programs were coded as 1, while states without such restrictions were coded as 0.

#### 2.3.4 Covariates

The study adjusted for demographic characteristics of individuals admitted to treatment centers at the state‐year level obtained through TEDS‐A, measured as proportions (including race and ethnicity, educational attainment, unemployment, homelessness, and arrest history), as well as state‐level factors such as the number of Medicaid beneficiaries, ACA Medicaid expansion status, the number of mental health or substance use treatment facilities, and prior authorization prohibition status on buprenorphine.

### 2.4 Statistical Analysis

First, the study employed a synthetic difference‐in‐differences (SDiD) approach developed by Arkhangelsky et al. [46] The SDiD method combines synthetic control method and DiD to provide a parallel pre‐exposure trend in the control units for the treated units. SDiD refines both approaches by using a weighted difference‐in‐differences estimator that matches units on pre‐exposure trends, thereby reducing reliance on the parallel trends assumption while accounting for inherent differences between treated and control units. Specifically, the SDiD method constructs a set of unit and time weights that minimize differences in trends during the pre‐exposure period. This approach is particularly useful when pre‐treatment differences are present and when treatment assignment may be correlated with unobserved confounders.

The study used the SDiD method with the binary treatment variables to estimate the average causal effect of funds used on MOUD admissions that takes the following form:

(1) $$\left({\hat{\tau }}^{\text{sdid}},\hat{\mu },\hat{\alpha },\hat{\beta }\right)=\arg\min_{\tau ,\mu ,\alpha ,\beta }\sum_{i=1}^{N}\sum_{t=1}^{T}{\hat{\omega_{i}}}^{\text{sdid}}{\hat{\lambda_{t}}}^{\text{sdid}}{\left(Y_{\mathrm{it}}-\mu -\alpha_{i}-\beta_{t}-\tau W_{\mathrm{it}}\right)}^{2}$$

where ${\hat{\tau }}^{\text{sdid}}$ is the estimated average treatment effect on the treated (ATT). $Y_{it}$ is the outcome variable for state i and year t. $W_{\mathrm{it}}$ is the binary treatment variable that equals 1 if state i is treated at year t, and 0 otherwise. μ is the constant. $\alpha_{i}$ is the state fixed effects that capture the unobserved time‐invariant differences between states. $\beta_{t}$ is the year fixed effects that capture the differences across years.

${\hat{\omega }}^{\text{sdid}}$ is the unit weights, which are the weights in synthetic control, to minimize the differences between the control units and the treated units in their pre‐treatment outcomes. ${\hat{\lambda }}^{\text{sdid}}$ is the weights applied to pre‐treatment periods to balance out less relevant years on the outcomes between treated and control states. The study applied block bootstrap methods for inference to relax assumptions on equal variance or equal time weights in treated and control groups.

#### 2.4.1 TWFE DDD

Second, the study employed a two‐way fixed effects (TWFE) difference‐in‐differences‐in‐differences (DDD) model to evaluate the heterogeneous effect of the funds between states that restrict and states that allow such supplantation. The general form of the DDD is below:

(2) $$\begin{array}{c} Y_{\mathrm{it}}=\beta_{7}\left(\text{Trea}t_{i}\times \mathrm{Pos}t_{t}\times \text{NoSupplan}t_{i}\right)+\text{Double Interactions}+\alpha_{i}+\tau_{t}+X_{\mathrm{it}}\gamma +\varepsilon_{\mathrm{it}} \end{array}$$

where $\beta_{7}$ is the DDD estimator that estimates the difference in the effect of the funding between states with non‐supplantation rules and states that permit supplantation. $\alpha_{i}$ is the state fixed effects and $\tau_{t}$ is the year fixed effects.

In both models, the study tested the parallel trends assumption, which is whether control units and treated units exhibited parallel trends in outcomes prior to treatment. The evidence of parallel trends assumptions and potential dynamic effects was presented using event studies. Standard errors were clustered at the state level. All statistical analyses were conducted in Stata version 18, and *p* < 0.10 was used for statistical significance.

### 2.5 Sensitivity Analyses

The study conducted several sensitivity analyses to ensure the robustness of results. Full model specifications and detailed results are provided in the Supporting Information.

#### 2.5.1 Continuous Treatment Variable

The continuous treatment variables measured Treatment bucket and MOUD‐specific allocations as a proportion of total funds received by the state, and as an absolute amount per capita, to evaluate the dose–response effect of funding. The use of the continuous measure is to capture whether variation in treatment intensity yields different effects on outcomes. Because the KFF tracker combined funding information for 2022 and 2023, this study applied the same proportional and per‐capita measures to each post‐treatment year.

#### 2.5.2 Drop Year 2020

Considering the restrictive access to treatment centers during the COVID‐19 pandemic, the study examined results after dropping year 2020 from the analysis.

#### 2.5.3 Controlling for Other SUD/OUD Funding

While opioid abatement funds provide an important financial resource for addressing the opioid epidemic, other federal funding for SUD and OUD may still influence MOUD utilization. This is particularly important as Lindenfeld and coauthors found that abatement funds are insufficient in scale to replace federal SUD funding [47]. To assess the robustness of the findings, the study included sensitivity analyses using the SDiD and TWFE DDD models that adjusted for state‐year variation in received federal SUD/OUD funding.

## 3 Results

During 2022–2023, 33 states in the analysis committed or spent abatement funds within the Treatment bucket, and among these, 31 states allocated funds to MOUD‐specific programs with the exceptions of Alabama and Maryland (Table 1).

**TABLE 1:** States status on opioid abatement fund in 2022–2023.

States committed or spent funds from treatment bucket	Total funds committed or spent from treatment bucket ($ in thousands)	Percentage of funds committed or spent from treatment bucket	States with no funds committed or spent from treatment bucket	States committed or spent funds on MOUD strategy	Total funds committed or spent on MOUD ($ in thousands)	Percentage of funds committed or spent on MOUD	Proportion of general treatment funds allocated to MOUD	States with no funds committed or spent on MOUD
Alabama	1500	15.00%	Alaska	Arkansas	1341	3.52%	11.82%	Alabama
Arkansas	11,341	29.79%	District of Columbia	California	7699	3.50%	68.51%	Alaska
California	11,237	5.11%	Hawaii	Colorado	10,977	17.77%	60.35%	District of Columbia
Colorado	18,187	29.44%	Louisiana	Connecticut	228	9.58%	100.00%	Hawaii
Connecticut	228	9.58%	Mississippi	Florida	18,000	10.13%	90.00%	Louisiana
Florida	20,000	11.26%	Nebraska	Georgia	8000	32.02%	100.00%	Maryland
Georgia	8000	32.02%	New Mexico	Illinois	18,000	20.85%	60.00%	Mississippi
Illinois	30,000	34.75%	Ohio	Indiana	7509	10.84%	95.87%	Nebraska
Indiana	7833	11.30%	Texas	Iowa	48	7.69%	90.54%	New Mexico
Iowa	53	8.49%		Kansas	4613	33.53%	88.50%	Ohio
Kansas	5212	37.89%		Kentucky	25,805	55.96%	98.30%	Texas
Kentucky	26,250	56.93%		Maine	3000	33.06%	100.00%	
Maine	3000	33.06%		Massachusetts	6349	16.02%	100.00%	
Maryland	7786	22.63%		Michigan	10,145	11.61%	54.83%	
Massachusetts	6349	16.02%		Minnesota	6158	23.54%	89.20%	
Michigan	18,501	21.18%		Missouri	3783	16.84%	90.97%	
Minnesota	6903	26.39%		Nevada	4125	39.54%	89.68%	
Missouri	4159	18.51%		New Hampshire	7222	17.59%	91.61%	
Nevada	4600	44.09%		New Jersey	1938	19.55%	100.00%	
New Hampshire	7883	19.20%		New York	43,650	11.51%	98.64%	
New Jersey	1938	19.55%		North Carolina	23,920	22.94%	76.03%	
New York	44,250	11.67%		North Dakota	1086	13.57%	100.00%	
North Carolina	31,462	30.17%		Pennsylvania	25,053	19.47%	72.05%	
North Dakota	1086	13.57%		Rhode Island	3607	11.72%	85.74%	
Pennsylvania	34,772	27.03%		South Dakota	73	5.86%	66.70%	
Rhode Island	4207	13.67%		Tennessee	12,156	37.99%	99.92%	
South Dakota	110	8.78%		Utah	2800	22.75%	73.68%	
Tennessee	12,166	38.02%		Vermont	2940	33.15%	100.00%	
Utah	3800	30.87%		Washington	12,338	14.85%	52.31%	
Vermont	2940	33.15%		Wisconsin	8384	14.15%	37.27%	
Washington	23,586	28.39%		Wyoming	1544	37.45%	100.00%	
Wisconsin	22,494	37.97%						
Wyoming	1544	37.45%						

*Note:* Arizona, Delaware, Idaho, Oregon, South Carolina, Montana, Oklahoma, Virginia, and West Virginia were removed from the analysis due to inconsistent data reporting in the Treatment Episode Data Set–Admissions (TEDS‐A) from 2016 to 2023.

Abbreviation: MOUD, medications for opioid use disorder.

*Source:* KFF Opioid Abatement Funding Tracker 2022–2023 (https://kffhealthnews.org/download‐the‐data‐opioid‐settlement‐expenditures/).

The share of total abatement funds allocated to the Treatment bucket varied substantially across states, ranging from 5% in California to 57% in Kentucky. Eight states allocated all Treatment funds exclusively to MOUD‐specific programs, including Connecticut, Georgia, Maine, Massachusetts, New Jersey, North Dakota, Vermont, and Wyoming. In contrast, Arkansas allocated the smallest proportion of Treatment funds to MOUD‐specific programs (12%).

### 3.1 Descriptive

#### 3.1.1 Funds on Treatment Bucket

States that committed to or spent funds from the Treatment bucket exhibited significantly higher SUD admission rates per 100,000 population (*p* < 0.001), higher MOUD admission rates per 100,000 population (*p* < 0.001), and a higher proportion of MOUD admissions among all SUD admissions (*p* = 0.02) compared to non‐allocating states (Table S1). These states also differed demographically. They had a higher proportion of non‐Hispanic White individuals among admissions (*p* < 0.001), whereas non‐allocating states had a higher proportion of admissions identifying as non‐Hispanic Black (*p* = 0.005) and non‐Hispanic other races (including non‐Hispanic Asian, native Hawaiian or other pacific Islander, Alaska Native, American Indian, other single race, and two or more races) (*p* < 0.001). In addition, allocating states had fewer admissions among individuals with less than high school education (*p* = 0.02) and more admissions with college education (*p* < 0.001). Allocating states also, on average, had a greater number of mental health or substance treatment facilities (*p* < 0.001).

#### 3.1.2 Funds on MOUD Strategy

States that committed or spent funds specifically to MOUD strategy had higher SUD admission rates per 100,000 population (*p* = 0.02) (Table S1). These states also had a higher proportion of non‐Hispanic White admissions (*p* < 0.001), and lower proportions of non‐Hispanic Black (*p* < 0.001) and non‐Hispanic other races (*p* = 0.008). Additionally, these states had higher proportions of admissions among individuals with some college education (*p* < 0.001) and college graduate (*p* = 0.001) and fewer with less than high school education (*p* < 0.001). They also had a higher proportion of unemployed admissions (*p* = 0.02), and more Medicaid beneficiaries (*p* = 0.02) and treatment facilities (*p* < 0.001).

### 3.2 SDiD Analysis

#### 3.2.1 Full Sample

Using the SDiD method on all admissions, event‐study plots indicated that pre‐treatment trends were generally parallel between treated and comparison states for both the Treatment bucket and MOUD‐specific allocations (Figure 1), supporting the identifying assumption.

**FIGURE 1:**
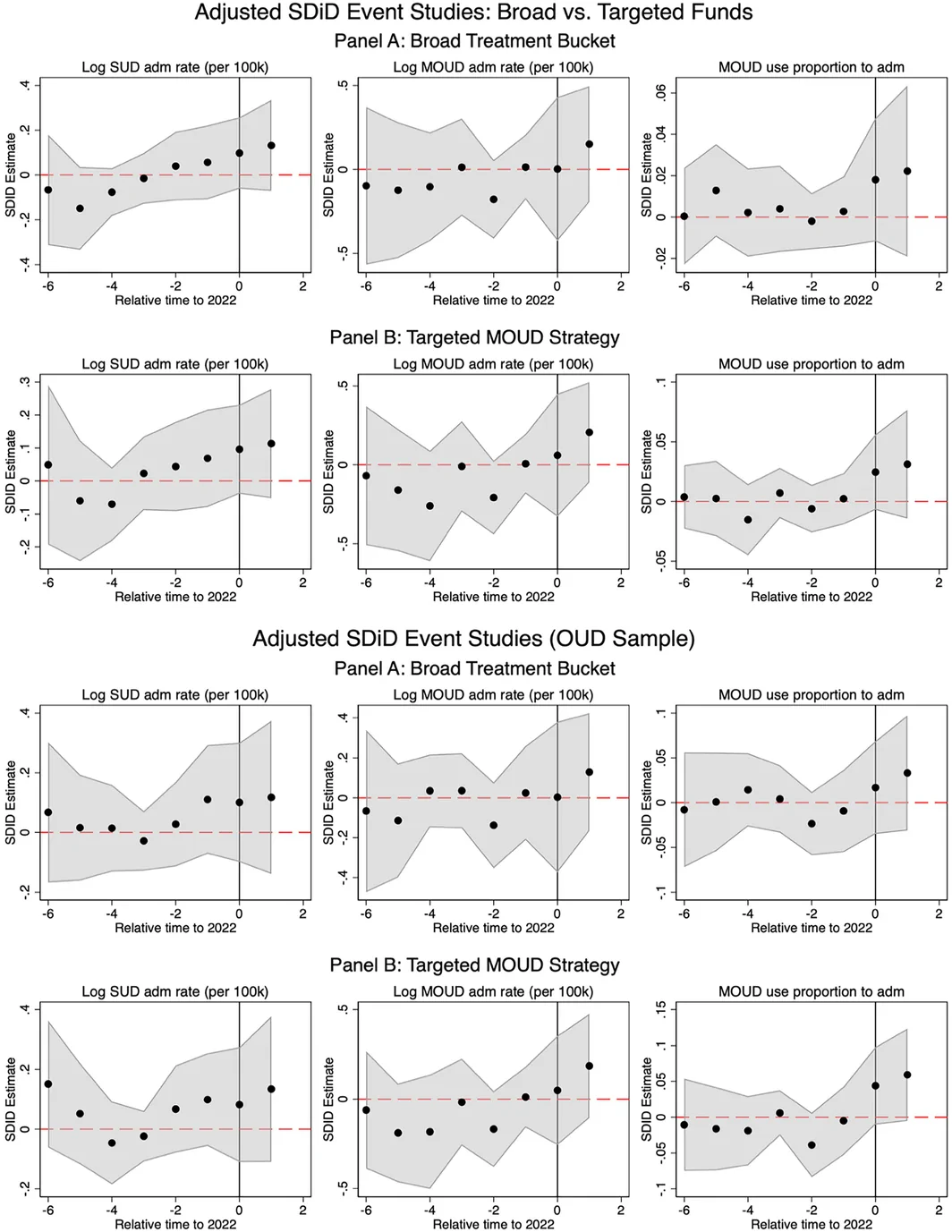
Event study and trajectory of the synthetic difference‐in‐differences (SDiD) analysis. adm, admission; MOUD, medications for opioid use disorder; OUD, opioid use disorder; SUD, substance use disorder.

#### 3.2.2 Funds on Treatment Bucket

SDiD estimates suggested that allocating funds to the Treatment bucket marginally increased SUD admission rates by 13.2% (*p* = 0.06) compared to states that did not allocate funds (Table 2, Panel A‐1). However, spending from the Treatment bucket had no significant effects on MOUD use admission rates or the proportion of admissions receiving MOUD.

**TABLE 2:** Main results using synthetic difference‐in‐differences.

Panel A‐1: Full sample	Committed or spent funds from treatment bucket						Committed or spent funds on MOUD strategy					
	Log SUD admission rate per 100,000 population		Log MOUD use rate per 100,000 population		MOUD use proportion		Log SUD admission rate per 100,000 population		Log MOUD use rate per 100,000 population		MOUD use proportion	
Opioid abatement funding	0.101	0.124*	0.168	0.126	0.020	0.010	0.052	0.078	0.165	0.128	0.036**	0.017
	(0.075)	(0.066)	(0.163)	(0.187)	(0.016)	(0.020)	(0.063)	(0.070)	(0.152)	(0.170)	(0.017)	(0.017)
Covariates	No	Yes	No	Yes	No	Yes	No	Yes	No	Yes	No	Yes
Pre‐treatment mean	6.374	6.374	3.716	3.716	0.120	0.120	6.356	6.356	3.648	3.648	0.114	0.114
Relative % change	10.70%	13.20%	18.30%	13.40%	16.80%	8.10%	5.40%	8.20%	18.00%	13.70%	31.40%	15.20%
Observations	336	336	336	336	336	336	336	336	336	336	336	336

Panel A‐2: OUD sample	Log SUD admission rate per 100,000 population		Log MOUD use rate per 100,000 population		MOUD use proportion		Log SUD admission rate per 100,000 population		Log MOUD use rate per 100,000 population		MOUD use proportion	
Opioid abatement funding	0.154	0.188	0.141	0.135	0.030	0.004	0.085	0.146	0.143	0.103	0.078**	0.036
	(0.110)	(0.115)	(0.138)	(0.176)	(0.033)	(0.036)	(0.092)	(0.111)	(0.122)	(0.142)	(0.039)	(0.026)
Covariates	No	Yes	No	Yes	No	Yes	No	Yes	No	Yes	No	Yes
Pre‐treatment mean	5.223	5.223	3.556	3.556	0.258	0.258	5.190	5.190	3.489	3.489	0.252	0.252
Relative % change	16.60%	20.70%	15.10%	14.40%	11.50%	1.50%	8.80%	15.70%	15.40%	10.80%	31.10%	14.10%
Observations	336	336	336	336	336	336	336	336	336	336	336	336

*Note:* The analyses used synthetic difference‐in‐differences estimators. Sample includes admissions to the substance treatment facilities. Regressions adjust for demographic characteristics of individuals admitted to treatment centers at the state‐year level including the proportion of race and ethnicity (Hispanic, non‐Hispanic White, non‐Hispanic Black, and non‐Hispanic other races, including Asian, native Hawaiian or other pacific Islander, Alaska Native, American Indian, other single race, and two or more races), educational attainment (less than high school, high school graduate, some college, and college graduate), unemployment, homelessness, and arrest history, as well as state‐level factors such as the number of Medicaid beneficiaries, ACA Medicaid expansion status, the number of mental health or substance use treatment facilities, and prior authorization prohibition status on buprenorphine. Year and state fixed effects are included, and robust standard errors are clustered at the state level. Standard errors in parentheses. *p < 0.10; **p < 0.05.

Abbreviations: MOUD, medications for opioid use disorder; OUD, opioid use disorder; SUD, substance use disorder.

*Source:* Treatment episode data set–admissions (TEDS‐A) 2016–2023.

#### 3.2.3 Funds on MOUD Strategy

Spending funds on MOUD‐specific strategies did not lead to statistically significant effects on SUD admissions, MOUD use admissions, or the proportion of admissions receiving MOUD (Table 2, Panel A‐1).

### 3.3 SDID Analysis

#### 3.3.1 OUD Sample

To focus on the population most directly targeted by MOUD expansion, the study further restricted the sample to admissions with OUD. Event‐study results continued to support the parallel trends assumption in this restricted sample (Figure 1).

SDiD estimates showed no statistically significant effects of either Treatment or MOUD‐specific allocations on SUD admissions, MOUD use admissions, or the proportion of admissions receiving MOUD (Table 2, Panel A‐2).

### 3.4 Treatment Heterogeneity by Supplantation Status

To examine heterogeneity in treatment effects, the study estimated a DDD model that interacted funding allocation with state‐level supplantation restrictions. Event‐study results indicated parallel pre‐treatment trends in the full sample and the OUD sample across states with and without supplantation restrictions (Figure 2). Cell composition showed that when the treatment variable was spending on the Treatment bucket, the Control and No‐Supplantation cell included only Washington DC; when the treatment variable was spending on MOUD‐specific programs, this cell included Washington DC and Maryland.

**FIGURE 2:**
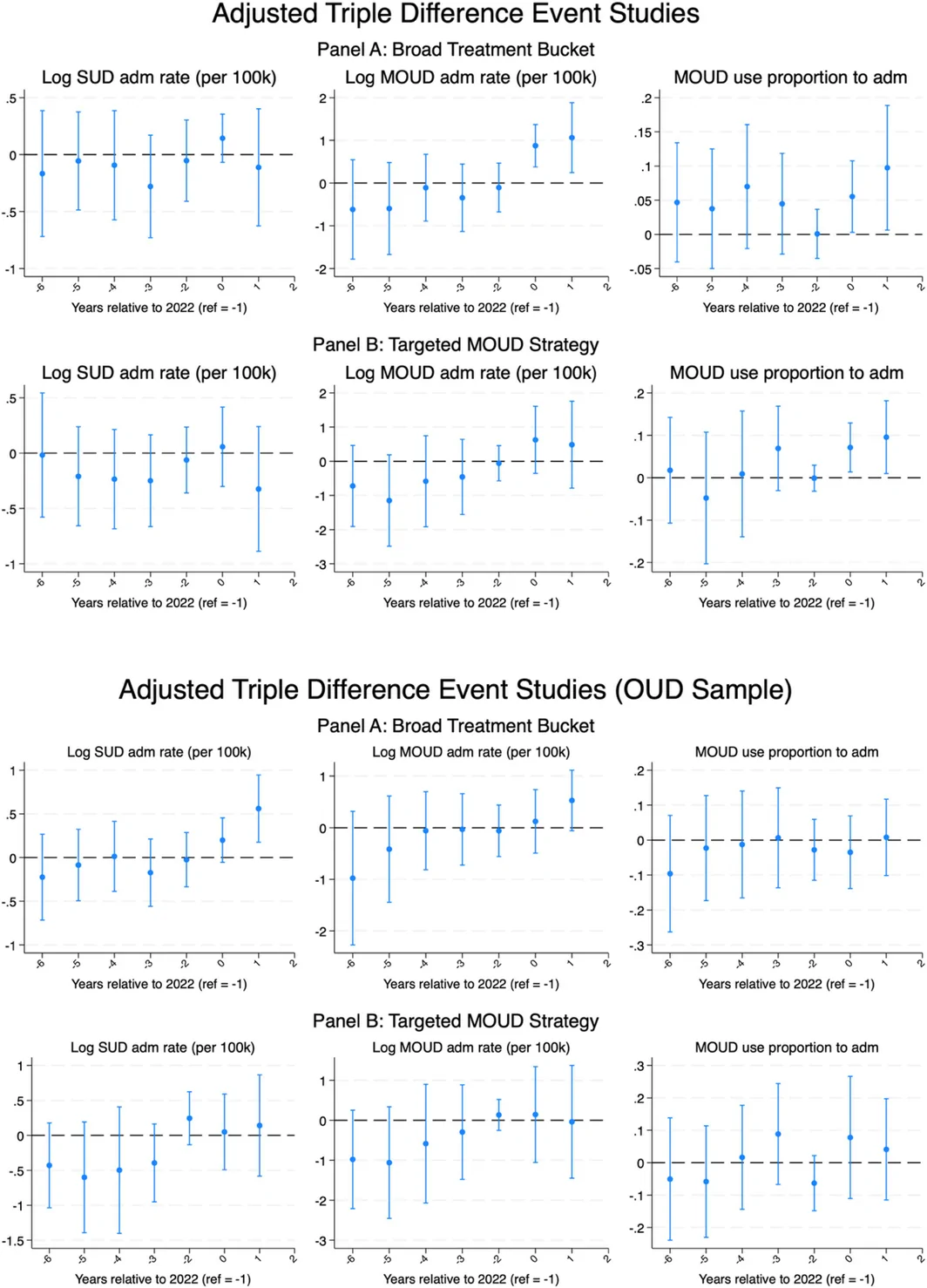
Event study of the two‐way fixed effects difference‐in‐differences‐in‐differences (TWFE DDD) analysis. adm, admission; MOUD, medications for opioid use disorder; OUD, opioid use disorder; SUD, substance use disorder.

#### 3.4.1 Funds on Treatment Bucket

In the full sample, the effect of the Treatment bucket allocation on MOUD admission rates was 232.7% (*p* = 0.009) larger in states that prohibited supplantation than in states that allowed it (Table 3, Panel A‐1). No statistically significant effects were observed in states that allow supplantation.

**TABLE 3:** Heterogenous effect analysis by state supplantation status.

Panel A‐1: Full sample	Committed or spent funds from treatment bucket			Committed or spent funds on MOUD strategy		
	Log SUD admission rate per 100,000 population	Log MOUD use rate per 100,000 population	MOUD use proportion	Log SUD admission rate per 100,000 population	Log MOUD use rate per 100,000 population	MOUD use proportion
Opioid abatement funding × post × not allow supplantation	0.137	1.202***	0.038	−0.006	0.947*	0.075*
	(0.230)	(0.436)	(0.031)	(0.264)	(0.505)	(0.039)
Opioid abatement funding × post	0.135	0.104	0.018	0.075	0.139	0.023
	(0.121)	(0.267)	(0.024)	(0.121)	(0.245)	(0.022)
Post × not allow supplantation	−0.071	−1.260***	−0.074**	0.072	−0.978**	−0.106***
	(0.218)	(0.394)	(0.030)	(0.251)	(0.436)	(0.034)
Covariates	Yes	Yes	Yes	Yes	Yes	Yes
Pre‐treatment mean	6.747	4.576	0.192	6.661	4.400	0.180
Relative % change	14.60%	232.70%	19.50%	−0.60%	157.70%	41.60%
Observations	336	336	336	336	336	336
Adj. *R* ^2^	0.905	0.895	0.811	0.904	0.895	0.815

Panel A‐2: OUD sample	Log SUD admission rate per 100,000 population	Log MOUD use rate per 100,000 population	MOUD use proportion	Log SUD admission rate per 100,000 population	Log MOUD use rate per 100,000 population	MOUD use proportion
Opioid abatement funding × post × not allow supplantation	0.463***	0.569*	0.008	0.386*	0.521	0.070
	(0.152)	(0.314)	(0.045)	(0.219)	(0.471)	(0.071)
Opioid abatement funding × post	0.001	0.106	0.046	−0.048	0.147	0.063*
	(0.138)	(0.189)	(0.038)	(0.135)	(0.184)	(0.037)
Post × not allow supplantation	−0.383**	−0.639**	−0.057	−0.280	−0.566	−0.112*
	(0.156)	(0.278)	(0.040)	(0.225)	(0.370)	(0.061)
Covariates	Yes	Yes	Yes	Yes	Yes	Yes
Pre‐treatment mean	5.773	4.420	0.340	5.671	4.259	0.326
Relative % change	58.90%	76.70%	2.40%	47.20%	68.40%	21.40%
Observations	336	336	336	336	336	336
Adj. *R* ^2^	0.910	0.905	0.844	0.909	0.905	0.849

*Note:* The analyses used two‐way fixed effects difference‐in‐differences‐in‐differences estimators. Sample includes admissions to the substance treatment facilities. Regressions adjust for demographic characteristics of individuals admitted to treatment centers at the state‐year level including the proportion of race and ethnicity (Hispanic, non‐Hispanic White, non‐Hispanic Black, and non‐Hispanic other races, including Asian, native Hawaiian or other pacific Islander, Alaska Native, American Indian, other single race, and two or more races), education attainment (less than high school, high school graduate, some college, and college graduate), unemployment, homelessness, and arrest history, as well as state‐level factors such as the number of Medicaid beneficiaries, ACA Medicaid expansion status, the number of mental health or substance use treatment facilities, and prior authorization prohibition status on buprenorphine. Year and state fixed effects are included, and robust standard errors are clustered at the state level. Standard errors in parentheses. *p < 0.10, **p < 0.05, ***p < 0.01.

Abbreviations: MOUD, medications for opioid use disorder; OUD, opioid use disorder; SUD, substance use disorder.

*Source:* Treatment Episode Data Set–Admissions (TEDS‐A) 2016–2023.

These patterns persist in the OUD‐restricted sample (Table 3, Panel A‐2). In non‐supplanting states, allocations to the Treatment bucket increased SUD admission rates by 58.9% (*p* = 0.004) and increased MOUD admission rates by 76.7% (*p* = 0.08), with no significant effects in supplantation states.

#### 3.4.2 Funds on MOUD Strategy

When analyzing funds specifically targeted MOUD strategies in the full sample, non‐supplanting states had larger increases in MOUD use admission rates by 157.7% (*p* = 0.07) and raised the proportion of MOUD admissions by 7.5 percentage points, which represent a 41.6% relative increase (*p* = 0.06) (Table 3, Panel A‐1).

Among OUD admissions, MOUD‐specific funding increased SUD admission rates by 47.2% (*p* = 0.09) in non‐supplanting states than in states that allowed it (Table 3, Panel A‐2). States that allowed supplantation also experienced a marginal increase in the proportion of admissions receiving MOUD by 6.3 percentage points (*p* = 0.09), although the differences were not significant compared to non‐supplanting states.

### 3.5 Sensitivity Analyses

Treating funding allocation as a continuous measure (proportion of funds allocated to Treatment bucket or MOUD) attenuated the estimated effects, yielding no statistically significant effects across outcomes (Table S2, Panel A‐1). When funding was measured as absolute dollars per capita, the results remained insignificant, though the point estimates for SUD and MOUD admission rates turned negative (Table S2, Panel A‐2).

Second, excluding the year 2020 strengthens the estimated effects for the Treatment bucket. In this specification, Treatment bucket allocation led to a marginally significant increase in the SUD admission rates by 21.10% (*p* = 0.09) (Table S2, Panel A‐3).

Lastly, results were robust when additionally adjusting for state‐level SUD/OUD funding per capita in both models, with estimates remaining generally consistent with those from the previous specifications (Tables S3 and S4).

## 4 Discussion

This study provides early causal evidence on whether opioid abatement funds allocated to treatment programs for addressing the opioid crisis have translated into expanded MOUD utilization. As indicated by the descriptive results, states that allocated abatement funding to treatment or MOUD‐specific programs differed systematically from states that did not. States that allocated opioid abatement funds generally differed from non‐allocating states in treatment admissions' racial and ethnic and educational composition, had higher levels of SUD treatment utilization, and more mental health or substance treatment facilities. These differences suggest that states entered the period of abatement fund implementation with different substance use treatment utilization patterns, needs, and service capacity.

In the main analysis, the study found that allocation of abatement funds to the general Treatment bucket modestly increases overall SUD treatment admissions, but no significant increase in MOUD utilization, neither in the full sample nor among admissions with identified opioid use. Allocations directed specifically at MOUD programs showed no detectable effect on any outcome. These results suggest that treatment‐oriented abatement investments may have begun to increase overall engagement with substance use treatment but were not yet sufficient to produce measurable changes in MOUD utilization during the early period of implementation. The absence of detectable effects for MOUD‐specific allocations warrants particular attention. One likely explanation is that MOUD is increasingly delivered through channels that TEDS‐A does not capture, including office‐based prescribing with pharmacy dispensing, telehealth, and emergency department [48]. Elimination of the X‐waiver requirement in late 2022 further shifted prescribing toward primary care providers during the post‐period [49]. Yet, this removal of a prescribing barrier did not translate into gains in patient‐level buprenorphine access [49], suggesting that expanding capacity alone may be insufficient to increase utilization in the short term.

The limited average effects may reflect the substantial heterogeneity in states' treatment systems, funding decisions, and implementation of abatement resources. The null effects for proportional or per‐capita allocations may reflect that these measures capture funding magnitude rather than implementation strategy. States that allocated funds already had higher SUD admission rates and more treatment facilities, suggesting more established treatment systems in which early additional funding may have had limited marginal effects on treatment capacity or admissions.

The exploratory analysis further examined whether state supplantation policies lead to heterogeneity in the relationship between abatement funding and treatment outcomes. Effects of funding allocation were larger in non‐supplanting states, with increased general treatment utilization among the OUD sample, whereas states that allow supplantation show no measurable differences. These findings suggest that requiring abatement funds to supplement, rather than replace existing expenditures, may generate greater net increases in resources available for treatment. Few jurisdictions both had no allocated funds to treatment and adopted non‐supplantation policies. Therefore, the supplantation findings should be viewed as suggestive rather than confirmatory and warrant further evaluation.

Taken together, the study examines whether opioid abatement funding influences MOUD utilization, while accounting for the substantial differences across states in treatment capacity, funding choices, and fiscal policies that shape how these resources are deployed. The findings highlight that the effectiveness of opioid abatement funding may depend not only on the amount or target of spending, but also on how new resources interact with existing treatment systems and state financing policies. Current allocation structures of the abatement funds across states and localities remain fragmented, with variation in oversight, governance, and restrictions on fund use [22, 50]. This fragmentation complicates both cross‐state comparisons and rigorous evaluation of whether funds achieve their intended outcomes. Moreover, expanding MOUD access requires more than increasing financial investment alone, but requires long‐term capacity building. Structural and socioeconomic barriers, including transportation, housing instability, provider stigma, and limited clinician knowledge of MOUD, continue to constrain utilization [16, 51, 52, 53, 54]. The prioritization of funded programs at the state and local level plays a critical role in determining the effectiveness of these investments. These factors may help explain the attenuated effects observed for both the general Treatment bucket and MOUD‐specific funding in this analysis, reflecting challenges in both implementation and measurement. In addition, the relatively short post‐intervention period may limit the ability to detect longer‐term effects on treatment outcomes. Because abatement funds are expected to be distributed over an 18‐year horizon, their full impact may unfold gradually over time. Nevertheless, these early findings provide useful preliminary evidence. Continued monitoring of abatement fund allocation, implementation, and treatment outcomes will be important as states move beyond the early years of abatement spending. Future research with longer follow‐up and sub‐state analyses, particularly in rural areas, can help clarify whether these investments expand access to evidence‐based treatment.

## 5 Limitations

This study has several limitations. First, TEDS‐A captures only individuals admitted to publicly funded treatment facilities, which may not represent the full population of individuals with OUD, particularly those not engaged in treatment. MOUD utilization is based on treatment plan documentation in TEDS‐A and may not reflect treatment intensity or adherence. Second, the KFF 2022–2023 funding tracker may not directly correspond to actual service delivery, may omit expenditures outside the reporting period or not captured during data collection, particularly localized spending [55], and reports 2022–2023 funding as a lump sum, limiting assessment of spending timing and potentially misclassifying the post‐treatment period. It also does not cleanly differentiate committed from spent funds. Moreover, state supplantation policies were derived from secondary sources and may not fully reflect their implementation or enforcement. Third, the DDD supplantation analysis relies on a small number of comparison states. These findings are therefore exploratory rather than confirmatory. Finally, although the SDiD approach helps account for pre‐treatment differences, causal identification relies on the validity of weighted parallel trends.

## Funding

The authors have nothing to report.

## Conflicts of Interest

The authors declare no conflicts of interest.

## Supporting information


**Data S1:** hesr70173‐sup‐0001‐Supinfo.docx.
**Table S1:** Descriptive statistics of treated and control groups by treatment variables.
**Table S2:** Sensitivity analyses results.
**Figure S1:** Event study of the SDiD adjusting for federal SUD/OUD funding.
**Figure S2:** Event study of the SDiD adjusting for federal SUD/OUD funding, OUD sample.
**Table S3:** SDiD analysis adjusting for federal SUD/OUD funding.
**Figure S3:** Event study of the TWFE DDD adjusting for federal SUD/OUD funding.
**Figure S4:** Event study of the TWFE DDD adjusting for federal SUD/OUD funding, OUD sample.
**Table S4:** TWFE DDD analysis adjusting for federal SUD/OUD funding.

## Data Availability

The data that support the findings of this study are available from public websites. These data were derived from the following resources available online: (1) Kaiser Family Foundation, URL https://kffhealthnews.org/download‐the‐data‐opioid‐settlement‐expenditures/; (2) Substance Abuse and Mental Health Services Administration, URL https://www.samhsa.gov/data/data‐we‐collect/teds‐treatment‐episode‐data‐set/datafiles/teds‐a‐2023.
