# Supplementary material for: The Effect of Opioid Abatement Funds on the Utilization of Medications for Opioid Use Disorder
Source: Health Serv Res. 2026 Nov 3;61(6):e70173. doi: 10.1111/1475-6773.70173 (PMC13728835; doi:10.1111/1475-6773.70173)
Supplement: Supplementary file 1 [file HESR-61-e70173-s001.docx]

**Supplemental Materials**

**Descriptive Statistics**

eTable 1. Descriptive statistics of treated and control groups by treatment variables

**Sensitivity Analyses**

Method

eTable 2. Sensitivity analyses results

Panel A-1: Continuous Treatment Variable (% of funds)

Panel A-2: Continuous Treatment Variable ($ funding per capita)

Panel A-3: Dropping Year 2020

Controlling for time-varying SUD/OUD funding capacity

eFigure 1. Event study of the SDiD adjusting for federal SUD/OUD funding

eFigure 2. Event study of the SDiD adjusting for federal SUD/OUD funding, OUD sample

eTable 3. SDiD analysis adjusting for federal SUD/OUD funding

eFigure 3. Event study of the TWFE DDD adjusting for federal SUD/OUD funding

eFigure 4. Event study of the TWFE DDD adjusting for federal SUD/OUD funding, OUD sample

eTable 4. TWFE DDD analysis adjusting for federal SUD/OUD funding

**References**

**State Level Analysis Descriptive Statistics**

eTable 1. Descriptive statistics of treated and control groups by treatment variables

|  | **States Committed or Spent Funds from Treatment Bucket** | | | | |  | **States Committed or Spent Funds on MOUD** | | | | |
| --- | --- | --- | --- | --- | --- | --- | --- | --- | --- | --- | --- |
| **Variables** | **Treated State-Year  (N = 264)** | | **Control State-Year  (N = 72)** | | **P-value** |  | **Treated State-Year  (N = 248)** | | **Control State-Year  (N = 88)** | | **P-value** |
|  | Average | SD | Average | SD |  |  | Average | SD | Average | SD |  |
| SUD admission rate per 100k population | 705.31 | 503.84 | 377.56 | 257.02 | <.001 |  | 674.32 | 445.60 | 524.49 | 557.39 | .02 |
| MOUD admission rate per 100k population | 104.18 | 154.79 | 37.67 | 47.97 | <.001 |  | 91.68 | 114.00 | 85.00 | 200.64 | .77 |
| Proportion of MOUD admissions | 0.12 | 0.11 | 0.09 | 0.10 | .02 |  | 0.12 | 0.11 | 0.11 | 0.11 | .29 |
| Proportion of Non-Hispanic White | 0.67 | 0.14 | 0.46 | 0.23 | <.001 |  | 0.67 | 0.14 | 0.48 | 0.22 | <.001 |
| Proportion of Non-Hispanic Black | 0.14 | 0.10 | 0.23 | 0.26 | .005 |  | 0.13 | 0.09 | 0.24 | 0.24 | <.001 |
| Proportion of Non-Hispanic Other Races | 0.07 | 0.10 | 0.17 | 0.22 | <.001 |  | 0.08 | 0.10 | 0.14 | 0.21 | .008 |
| Proportion of Hispanic | 0.11 | 0.09 | 0.14 | 0.16 | .13 |  | 0.11 | 0.09 | 0.12 | 0.15 | .88 |
| Proportion of Less Than High School | 0.25 | 0.05 | 0.28 | 0.08 | .02 |  | 0.25 | 0.05 | 0.28 | 0.07 | <.001 |
| Proportion of High School Graduate | 0.50 | 0.06 | 0.50 | 0.06 | .90 |  | 0.50 | 0.06 | 0.50 | 0.06 | .88 |
| Proportion of Some College | 0.19 | 0.05 | 0.18 | 0.05 | .15 |  | 0.20 | 0.05 | 0.17 | 0.06 | <.001 |
| Proportion of College Graduate | 0.06 | 0.02 | 0.04 | 0.01 | <.001 |  | 0.05 | 0.02 | 0.05 | 0.02 | .001 |
| Proportion of Unemployed | 0.44 | 0.14 | 0.42 | 0.20 | .29 |  | 0.45 | 0.14 | 0.40 | 0.18 | .02 |
| Proportion of Homeless | 0.17 | 0.10 | 0.19 | 0.13 | .14 |  | 0.17 | 0.09 | 0.19 | 0.15 | .24 |
| Proportion of Arrested | 0.09 | 0.05 | 0.10 | 0.09 | .21 |  | 0.09 | 0.05 | 0.10 | 0.08 | .57 |
| State's total population (in thousands) | 7247 | 7564 | 6152 | 8807 | .34 |  | 7358 | 7791 | 6040 | 7964 | .18 |
| State's number of Medicaid beneficiaries (in thousands) | 1830 | 2440 | 1382 | 1532 | .06 |  | 1868 | 2513 | 1355 | 1388 | .02 |
| State's number of mental health or substance treatment centers | 468 | 478 | 248 | 282 | <.001 |  | 474 | 489 | 272 | 279 | <.001 |

Source: Treatment Episode Data Set–Admissions (TEDS-A). Abbreviation: SUD, substance use disorder; MOUD, medications for opioid use disorder; SD, standard deviation. Welch’s t-test was used to compare variables between the Treated and Control groups, and the corresponding p-values were reported. Note: The number of observation is state-year from 2016-2023. Non-Hispanic other races include Asian, native Hawaiian or other pacific Islander, Alaska Native, American Indian, other single race, and two or more races. State population and Medicaid population were obtained from the University of Kentucky Center for Poverty Research. The number of mental health and substance use treatment facility at state level was extracted from the Census County Business Patterns datasets using the identification strategy described in prior studies, based on the North American Industry Classification System (NAICS) codes 623220 (residential mental health and substance abuse centers) and 621420 (outpatient mental health and substance abuse centers).^1–3^

**Sensitivity Analysis** **at the State Level**

Method

*TWFE DiD*

The study employed a Two-Way Fixed Effect (TWFE) Difference-in-Differences (DiD) model to assess the policy effect at the state level for sensitivity analyses. The general form of the DiD is below:

$$\begin{aligned} Y_{it}=\beta_{3}\left( \text{Treatment}_{i}\times\text{Post}_{t} \right)+\lambda X_{it}+\alpha_{i}+\beta_{t}+\epsilon_{it}\#\left( 1 \right) \end{aligned}$$

where $Y_{it}$ is the outcome variable for state $i$ and year $t$. $\beta_{3}$ is the parameter of interest that estimates the funds’ effects on state where states committed or spent abatement funds on the general Treatment bucket or MAT strategy. $\alpha_{i}$ is the unit fixed effects at the state level and $\beta_{t}$ is the year fixed effects. $\lambda X_{it}$ is the time-varying state level covariates.

The study tested the parallel trend assumption, which is whether control units and treated units exhibited parallel trends in outcomes prior to treatment. The evidence of parallel trend assumptions and potential dynamic effects were presented using event plots. Standard errors were clustered at the state level. All statistical analyses were conducted in Stata version 18, and p < 0.10 was presented for statistical significance.

eTable 2. Sensitivity analyses results

|  | **Committed or Spent Funds from Treatment Bucket** | | |  | **Committed or Spent Funds on MOUD Strategy** | | |
| --- | --- | --- | --- | --- | --- | --- | --- |
| **Panel A-1: Continuous Treatment Variable** | Log SUD admission rate per 100,000 population | Log MOUD use rate per 100,000 population | MOUD use proportion |  | Log SUD admission rate per 100,000 population | Log MOUD use rate per 100,000 population | MOUD use proportion |
| Opioid Abatement Funding (% of funds) | 0.284 | 0.611 | 0.035 |  | 0.249 | 0.257 | 0.030 |
|  | (0.263) | (0.740) | (0.054) |  | (0.284) | (0.837) | (0.052) |
|  |  |  |  |  |  |  |  |
| Covariates | Yes | Yes | Yes |  | Yes | Yes | Yes |
| Pre-Treatment Mean | 6.374 | 3.716 | 0.120 |  | 6.356 | 3.648 | 0.114 |
| Relative % Change | 32.90% | 84.20% | 29.50% |  | 28.20% | 29.20% | 25.90% |
| Observations | 336 | 336 | 336 |  | 336 | 336 | 336 |
| Adj. R-squared | 0.904 | 0.893 | 0.806 |  | 0.904 | 0.893 | 0.806 |
|  |  |  |  |  |  |  |  |
| **Panel A-2: Continuous Treatment Variable** | Log SUD admission rate per 100,000 population | Log MOUD use rate per 100,000 population | MOUD use proportion |  | Log SUD admission rate per 100,000 population | Log MOUD use rate per 100,000 population | MOUD use proportion |
| Opioid Abatement Funding ($ funding per capita) | -0.048 | -0.027 | 0.005 |  | -0.057 | -0.079 | 0.003 |
|  | (0.034) | (0.068) | (0.007) |  | (0.040) | (0.078) | (0.007) |
|  |  |  |  |  |  |  |  |
| Covariates | Yes | Yes | Yes |  | Yes | Yes | Yes |
| Pre-Treatment Mean | 6.374 | 3.716 | 0.120 |  | 6.356 | 3.648 | 0.114 |
| Relative % Change | -4.70% | -2.70% | 4.20% |  | -5.50% | -7.60% | 2.50% |
| Observations | 336 | 336 | 336 |  | 336 | 336 | 336 |
| Adj. R-squared | 0.905 | 0.893 | 0.807 |  | 0.905 | 0.894 | 0.806 |
|  |  |  |  |  |  |  |  |
| **Panel A-3: Dropping Year 2020** | Log SUD admission rate per 100,000 population | Log MOUD use rate per 100,000 population | MOUD use proportion |  | Log SUD admission rate per 100,000 population | Log MOUD use rate per 100,000 population | MOUD use proportion |
| Opioid Abatement Funding (binary) | 0.191* | 0.197 | 0.007 |  | 0.105 | 0.249 | 0.023 |
|  | (0.110) | (0.269) | (0.021) |  | (0.103) | (0.232) | (0.019) |
|  |  |  |  |  |  |  |  |
| Covariates | Yes | Yes | Yes |  | Yes | Yes | Yes |
| Pre-Treatment Mean | 6.407 | 3.724 | 0.120 |  | 6.390 | 3.653 | 0.114 |
| Relative % Change | 21.10% | 21.70% | 5.70% |  | 11.10% | 28.30% | 20.50% |
| Observations | 294 | 294 | 294 |  | 294 | 294 | 294 |
| Adj. R-squared | 0.897 | 0.890 | 0.806 |  | 0.895 | 0.890 | 0.808 |

Source: Treatment Episode Data Set–Admissions (TEDS-A) 2016-2023. Abbreviation: SUD, substance use disorder; MOUD, medications for opioid use disorder. Notes: The analyses used Two-Way Fixed Effects Difference-in-Differences estimator. Sample includes admissions to the substance treatment facilities. Regressions adjust for demographic characteristics of individuals admitted to treatment centers at the state-year level including the proportion of race and ethnicity, education attainment, unemployment, homelessness, and arrest history, as well as state-level factors such as the number of Medicaid beneficiaries, ACA Medicaid expansion status, the number of mental health or substance use treatment facilities, and prior authorization prohibition status on buprenorphine. Year and state fixed effects are included, and robust standard errors are clustered at the state level. Standard errors in parentheses. * p<0.10 ** p<0.05 *** p<0.01

Controlling for federal SUD and OUD funding capacity

Although the opioid abatement funding introduces a substantial new stream of revenue, states continue to receive concurrent federal funding, such as State Opioid Response grants and Substance Abuse Prevention and Treatment Block Grants. To assess whether the findings were influenced by variations in federal SUD/OUD funding across states and years, the study conducted state-level sensitivity analyses using SDiD and TWFE DDD models that additionally adjusted for federal SUD/OUD funding received per capita.

The study obtained year-by-year state funding data received from the Substance Abuse and Mental Health Services Administration (SAMHSA) via the Tracking Accountability in Government Grants System (TAGGS). Funding on SUD or OUD was identified using the following Assistance Listings:

- 93243: Substance Abuse and Mental Health Services Projects of Regional and National Significance
- 93788: State Opioid Response (SOR) Grants, and
- 93959: Block Grants for Prevention and Treatment of Substance Abuse (SAPT)

Using these data, the study calculated annual per capita SUD/OUD funding received for each state by dividing total funding received in each state-year by the corresponding state population.

eFigure 1. Event study of the SDiD adjusting for federal SUD/OUD funding

eFigure 2. Event study of the SDiD adjusting for federal SUD/OUD funding, OUD sample

eTable 3. SDiD analysis adjusting for federal SUD/OUD funding

|  | **Committed or Spent Funds from Treatment Bucket** | | | | | |  | **Committed or Spent Funds on MOUD Strategy** | | | | | |
| --- | --- | --- | --- | --- | --- | --- | --- | --- | --- | --- | --- | --- | --- |
| **Panel A-1: Full Sample** | Log SUD admission rate per 100,000 population | | Log MOUD use rate per 100,000 population | | MOUD use proportion | |  | Log SUD admission rate per 100,000 population | | Log MOUD use rate per 100,000 population | | MOUD use proportion | |
| Opioid Abatement Funding | 0.101 | 0.123* | 0.168 | 0.119 | 0.020 | 0.007 |  | 0.052 | 0.079 | 0.165 | 0.117 | 0.036** | 0.016 |
|  | (0.075) | (0.067) | (0.163) | (0.188) | (0.016) | (0.017) |  | (0.063) | (0.074) | (0.152) | (0.163) | (0.017) | (0.017) |
|  |  |  |  |  |  |  |  |  |  |  |  |  |  |
| Covariates | No | Yes | No | Yes | No | Yes |  | No | Yes | No | Yes | No | Yes |
| Pre-Treatment Mean | 6.374 | 6.374 | 3.716 | 3.716 | 0.120 | 0.120 |  | 6.356 | 6.356 | 3.648 | 3.648 | 0.114 | 0.114 |
| Relative % Change | 10.70% | 13.00% | 18.30% | 12.60% | 16.80% | 5.50% |  | 5.40% | 8.20% | 18.00% | 12.40% | 31.40% | 14.00% |
| Observations | 336 | 336 | 336 | 336 | 336 | 336 |  | 336 | 336 | 336 | 336 | 336 | 336 |
|  |  |  |  |  |  |  |  |  |  |  |  |  |  |
| **Panel A-2: OUD Sample** | Log SUD admission rate per 100,000 population | | Log MOUD use rate per 100,000 population | | MOUD use proportion | |  | Log SUD admission rate per 100,000 population | | Log MOUD use rate per 100,000 population | | MOUD use proportion | |
| Opioid Abatement Funding | 0.154 | 0.161 | 0.141 | 0.120 | 0.030 | 0.003 |  | 0.085 | 0.091 | 0.143 | 0.106 | 0.078** | 0.036 |
|  | (0.110) | (0.115) | (0.138) | (0.172) | (0.033) | (0.033) |  | (0.092) | (0.108) | (0.122) | (0.140) | (0.039) | (0.026) |
|  |  |  |  |  |  |  |  |  |  |  |  |  |  |
| Covariates | No | Yes | No | Yes | No | Yes |  | No | Yes | No | Yes | No | Yes |
| Pre-Treatment Mean | 5.223 | 5.223 | 3.556 | 3.556 | 0.258 | 0.258 |  | 5.190 | 5.190 | 3.489 | 3.489 | 0.252 | 0.252 |
| Relative % Change | 16.60% | 17.50% | 15.10% | 12.70% | 11.50% | 1.20% |  | 8.80% | 9.50% | 15.40% | 11.10% | 31.10% | 14.20% |
| Observations | 336 | 336 | 336 | 336 | 336 | 336 |  | 336 | 336 | 336 | 336 | 336 | 336 |

Source: Treatment Episode Data Set–Admissions (TEDS-A) 2016-2023. Abbreviations: SUD, substance use disorder; MOUD, medications for opioid use disorder; OUD, opioid use disorder. Notes: The analyses used Synthetic Difference-in-Differences-in-Differences estimators. Sample includes admissions to the substance treatment facilities. Regressions adjust for demographic characteristics of individuals admitted to treatment centers at the state-year level including the proportion of race and ethnicity, education attainment, unemployment, homelessness, and arrest history, as well as state-level factors such as the number of Medicaid beneficiaries, ACA Medicaid expansion status, the number of mental health or substance use treatment facilities, prior authorization prohibition status on buprenorphine, and received federal funding for substance use disorder and opioid use disorder per capita by state and year. Year and state fixed effects are included, and robust standard errors are clustered at the state level. Standard errors in parentheses. * p<0.10 ** p<0.05 *** p<0.01

eFigure 3. Event study of the TWFE DDD adjusting for federal SUD/OUD funding

eFigure 4. Event study of the TWFE DDD adjusting for federal SUD/OUD funding, OUD sample

eTable 4. TWFE DDD analysis adjusting for federal SUD/OUD funding

|  | **Committed or Spent Funds from Treatment Bucket** | | |  | **Committed or Spent Funds on MOUD Strategy** | | |
| --- | --- | --- | --- | --- | --- | --- | --- |
| **Panel A-1: Full Sample** | Log SUD admission rate per 100,000 population | Log MOUD use rate per 100,000 population | MOUD use proportion |  | Log SUD admission rate per 100,000 population | Log MOUD use rate per 100,000 population | MOUD use proportion |
| Opioid Abatement Funding x Post x Not Allow Supplantation | 0.119 | 1.156** | 0.041 |  | -0.021 | 0.912* | 0.077** |
|  | (0.233) | (0.441) | (0.031) |  | (0.261) | (0.509) | (0.038) |
|  |  |  |  |  |  |  |  |
| Opioid Abatement Funding x Post | 0.125 | 0.078 | 0.020 |  | 0.069 | 0.124 | 0.024 |
|  | (0.122) | (0.268) | (0.023) |  | (0.122) | (0.246) | (0.021) |
|  |  |  |  |  |  |  |  |
| Post x Not Allow Supplantation | -0.051 | -1.209*** | -0.077** |  | 0.088 | -0.942** | -0.108*** |
|  | (0.223) | (0.405) | (0.031) |  | (0.248) | (0.445) | (0.033) |
|  |  |  |  |  |  |  |  |
| Covariates | Yes | Yes | Yes |  | Yes | Yes | Yes |
| Pre-Treatment Mean | 6.747 | 4.576 | 0.192 |  | 6.661 | 4.400 | 0.180 |
| Relative % Change | 12.60% | 217.60% | 21.20% |  | -2.10% | 148.90% | 43.00% |
| Observations | 336 | 336 | 336 |  | 336 | 336 | 336 |
| Adj. R-squared | 0.905 | 0.896 | 0.811 |  | 0.904 | 0.896 | 0.816 |
|  |  |  |  |  |  |  |  |
| **Panel A-2: OUD Sample** | Log SUD admission rate per 100,000 population | Log MOUD use rate per 100,000 population | MOUD use proportion |  | Log SUD admission rate per 100,000 population | Log MOUD use rate per 100,000 population | MOUD use proportion |
| Opioid Abatement Funding x Post x Not Allow Supplantation | 0.436** | 0.504 | 0.013 |  | 0.360 | 0.459 | 0.075 |
|  | (0.163) | (0.311) | (0.048) |  | (0.221) | (0.457) | (0.074) |
|  |  |  |  |  |  |  |  |
| Opioid Abatement Funding x Post | -0.009 | 0.083 | 0.047 |  | -0.054 | 0.135 | 0.064* |
|  | (0.142) | (0.187) | (0.037) |  | (0.137) | (0.183) | (0.036) |
|  |  |  |  |  |  |  |  |
| Post x Not Allow Supplantation | -0.354** | -0.571** | -0.062 |  | -0.255 | -0.507 | -0.117* |
|  | (0.171) | (0.281) | (0.043) |  | (0.228) | (0.361) | (0.063) |
|  |  |  |  |  |  |  |  |
| Covariates | Yes | Yes | Yes |  | Yes | Yes | Yes |
| Pre-Treatment Mean | 5.773 | 4.420 | 0.340 |  | 5.671 | 4.259 | 0.326 |
| Relative % Change | 54.70% | 65.50% | 3.70% |  | 43.30% | 58.20% | 22.90% |
| Observations | 336 | 336 | 336 |  | 336 | 336 | 336 |
| Adj. R-squared | 0.910 | 0.906 | 0.844 |  | 0.910 | 0.907 | 0.849 |

Source: Treatment Episode Data Set–Admissions (TEDS-A) 2016-2023. Abbreviations: SUD, substance use disorder; MOUD, medications for opioid use disorder; OUD, opioid use disorder. Notes: The analyses used Two-Way Fixed Effects Difference-in-Differences-in-Differences estimators. Sample includes admissions to the substance treatment facilities. Regressions adjust for demographic characteristics of individuals admitted to treatment centers at the state-year level including the proportion of race and ethnicity, education attainment, unemployment, homelessness, and arrest history, as well as state-level factors such as the number of Medicaid beneficiaries, ACA Medicaid expansion status, the number of mental health or substance use treatment facilities, prior authorization prohibition status on buprenorphine, and received federal funding for substance use disorder and opioid use disorder per capita by state and year. Year and state fixed effects are included, and robust standard errors are clustered at the state level. Standard errors in parentheses. * p<0.10 ** p<0.05 *** p<0.01

**References**

1.	Lennon C, Maclean JC, Teltser K. Ridesharing and substance use disorder treatment. *J Health Econ*. 2025;99:102941. doi:10.1016/j.jhealeco.2024.102941

2.	Swensen ID. Substance-abuse treatment and mortality. *J Public Econ*. 2015;122:13-30. doi:10.1016/j.jpubeco.2014.12.008

3.	Deza M, Maclean JC, Solomon K. Local access to mental healthcare and crime. *J Urban Econ*. 2022;129:103410. doi:10.1016/j.jue.2021.103410
